# Notes on Health Resorts

**Published:** 1899-06-17

**Authors:** 


### The Institutional Workshop.

### NOTES ON HEALTH RESORTS.

### BUXTON.

Buxton is a little town in Derbyshire, standing upwards of 1,000 feet above the sea, and yet surrounded by hills much higher than itself. In fact, high as the town stands, it yet is in a valley, and thus is much protected by the hills and moors which extend for miles around. In the season it is a busy little place, and is a centre from which many interesting excursions can be made to all parts of the Peak district. Moreover, notwithstanding the large number of patients who annually resort to it for the sake of its waters and its bracing air, and of the crowds of pleasure seekers who go there for the sake of change and amusement, Buxton still remains a small town of not more than about 8,000 inhabitants; and in its smallness lies one of its advantages, for while it is in essence a watering-place, with all the shops and hotels, the gaiety and the life of a place in which money is freely spent by well-to-do people, it also, so far as its atmosphere and surroundings are concerned, remains in the country. Those who have had to wander for miles in search of open country when staying at popular health resorts which, from one cause or another, have grown into great residential towns, will understand what we mean when we say that it is an immense advantage, from the health seeker's point of view, that a health resort should remain small. Buxton again differs much from most of our English health resorts in regard to its elevation. The proximity of the sea on every side and the undoubted virtues of sea air have had their share in causing most of our watering-places to be located at the seaside, and, therefore, at a low altitude, whereas Buxton stands 1,000 feet above the sea level; and although, compared with some of the resorts abroad, this elevation is nothing very great, it is enough to give to Buxton quite a special kind of tonic influence upon the cases which go there. The researches which have been made during recent years in regard to the influence of mountain air upon anæmia go far to explain much of that bracing effect which is so marked a feature of the Buxton climate, and which exercises so beneficial an effect upon many of the invalids who resort to it year after year. The accompanying anæmia is one of the most serious complications of many of the cases of gout and rheumatism, and especially of the cases of rheumatoid arthritis, which resort to watering-places for relief, and there is much reason for believing that the well-known action of increased altitude in causing an improvement not only in the number of blood corpuscles but in the amount of hæmoglobin they contain has much to do with the tonic influence of Buxton in these cases. It is true that on descending from such elevated regions the improvement in chlorotic cases is to a certain extent lost, but it is not lost altogether; a certain balance remains to the good, and thus we find that for a considerable time after a "mountain cure" its good influence upon the blood remains.

To measure the benefits of change of air by the immediate effects which such change may be expected to have upon the economy is, however, but a narrow way of looking at the problem. Most invalids have within them a strong inherent tendency to recover if they do but get upon the right track, and when the improvement in the blood condition which results from residing in an elevated region enables the patient once again to take exercise, to live in the open air, and to lead a healthy life, to all of which he or she may, perhaps, have been unused for months before, the vicious circle of the invalid life is broken up, and the improvement in the blood, at first induced as a mere temporary effect of "altitude," becomes permanent. This is what is seen constantly when young anæmics go up into the mountains; and we can hardly avoid the conclusion that some of the tonic influence of mountain bath treatment upon enfeebled rheumatic and gouty patients has to do with the effect of the high altitude upon their blood condition, in addition to the more or less specific influence of the baths upon their ailments.

It is, however, on the virtues of its baths that Buxton has built up its reputation; and in these days of experimental medicine, when everything is on trial and when it is difficult, as indeed it often is, to ascertain how far the grounds upon which this place, that place, or the other is recommended are founded on theory or on fact, it is something for a place to be able to boast, as Buxton can, that it has been famous for the curative effects of its waters for hundreds of years, and that its repute in this respect has been the outcome, not of more or less theoretical opinions, but of plain practical experience by many generations of sufferers. It has to be confessed, however, that when we deal with these wonderful waters analytically, and endeavour to find out wherein their virtues lie, we do not find much to lay hold of beyond the fact that, weak as their mineralisation is, its character is much the same as that of other waters in other parts of the world which have also earned for themselves reputations quite as difficult to explain on chemical grounds as is that of Buxton.

Buxton water may be described as belonging to the class of simple thermal waters, or with equal accuracy as being a weakly mineralised earthy or calcareous water. The following is the result of one of the latest analyses by Dr. Otto Hehner :—

| | Grains per Imperial gallon. | | Grains per Imperial gallon. |
|---|---|---|---|
| Chloride of sodium... | 4·51717 | Carbonate of iron ... | 0·03709 |
| Sulphate of soda ... | 0·20203 | Carbonate of manganese ... ... ... | 0·00847 |
| Sulphate of potash ... | 0·66896 | Silica ... ... ... ... | 0·83769 |
| Sulphate of ammonium ... ... ... | 0·01564 | | |
| Sulphate of lime ... | 0·67364 | Total ... ... | ...21·13006 |
| Nitrate of lime ... | 0·25660 | Phosphoric acid ... | trace |
| Carbonate of lime ... | 9·18584 | Iodine ... ... ... | trace |
| Carbonate of magnesia ... ... ... | 4·72693 | Lithia ... ... ... | trace |

In addition to the above saline and other constituents, the water contains large quantities of free nitrogen gas, and the existence of the new elements, argon and helium, has recently been discovered.

The temperature of the water, as it flows into the natural bath, is 82 degrees Fahr., and at this temperature it is largely used for bathing purposes, the stream of fresh thermal water constantly flowing at a rate of from 150 to 300 gallons a minute. For many cases, however, a higher temperature than this is desirable, and for such patients a complete service of warm baths is provided, the natural water being heated to the required temperature by artificial means.

All the baths at Buxton are the property of the Duke of Devonshire. They are very well fitted up, and are provided with douches, with crane chairs for the use of helpless cripples, and with many of the hydrotherapeutic appliances which are met with in foreign bath-houses. Of late years the so-called Nauheim method for the treatment of heart disease by baths and resisted exercises has been introduced; and, take it altogether, Buxton has shown a progressive spirit in the management of its baths which speaks well for its future.

As to the place which Buxton takes in therapeutics it may broadly be said that its repute mainly depends upon its efficacy in the relief of chronic gouty and rheumatic conditions, and in stiff and painful cases of rheumatoid arthritis, but it also is of much service in many cases of gouty dyspepsia, of "torpid liver," and other cases in which exercise, fresh air, and a free diuresis are indicated, and especially in anæmia arising from city life. How it is that such weak waters as of Buxton, Wildbad, Ragatz-Pfaefers, and others of like character effect the cures which they seem to produce it is very difficult to say, but it is a matter of considerable interest to find that these three celebrated baths, each of which has built up a reputation for itself quite independent of the others, have certain characteristics in common. They all stand at a considerable elevation and are surrounded by hills, they all have waters which are so weakly mineralised as to be not unfitly classed among the simple thermal waters, and in each case the water is charged with nitrogen gas. The latter is perhaps a trivial point, as we can hardly attribute therapeutic efficiency to the amount of nitrogen contained in these waters; still it is interesting as a point of similarity between waters which have become famous for their efficacy in a similar class of cases. In addition to the thermal waters, Buxton has a very weak chalybeate spring.

